# Curing Effects on Interfacial Adhesion between Recycled Carbon Fiber and Epoxy Resin Heated by Microwave Irradiation

**DOI:** 10.3390/ma11040493

**Published:** 2018-03-26

**Authors:** Yuichi Tominaga, Daisuke Shimamoto, Yuji Hotta

**Affiliations:** National Institute of Advanced Industrial Science and Technology (AIST), Shimosidami, Moriyama-ku, Nagoya 463-8560, Japan; d-shimamoto@aist.go.jp (D.S.); y-hotta@aist.go.jp (Y.H.)

**Keywords:** interfacial adhesion, recycled carbon fiber, microwave heating, epoxy curing

## Abstract

The interfacial adhesion of recycled carbon fiber (CF) reinforced epoxy composite heated by microwave (MW) irradiation were investigated by changing the curing state of the epoxy resin. The recycled CF was recovered from the composite, which was prepared by vacuum-assisted resin transfer molding, by thermal degradation at 500 or 600 °C. Thermogravimetric analysis showed that the heating at 600 °C caused rough damage to the CF surface, whereas recycled CF recovered at 500 °C have few defects. The interfacial shear strength (IFSS) between recycled CF and epoxy resin was measured by a single-fiber fragmentation test. The test specimen was heated by MW after mixing the epoxy resin with a curing agent or pre-curing, in order to investigate the curing effects on the matrix resin. The IFSSs of the MW-irradiated samples were significantly varied by the curing state of the epoxy resin and the surface condition of recycled CF, resulting that they were 99.5 to 131.7% of oven heated samples Furthermore, rheological measurements showed that the viscosity and shrinking behaviors of epoxy resin were affected based on the curing state of epoxy resin before MW irradiation.

## 1. Introduction

Carbon fiber-reinforced plastic (CFRP) is attracting attention due to its excellent mechanical and light-weight properties [1,2,3,4,5,6,7]. Because the production of CFRPs has increased, there is increasing environmental and economic awareness for the need to recycle CFRP waste. Typically, CFRP waste includes various CFRPs, such as expired prepregs, manufacturing cut-offs, testing materials, production tools, and end-of-life components. Furthermore, recycled CFs can be recovered from CFRP wastes by various recycling processes, such as thermal degradation [8,9], chemical decomposition [10,11], and superheated steam [12], due to which the recycled CF has some surface defects, such as pitting, residual matrix, and char. In addition, all recycling processes remove the sizing agent from CF surfaces; hence, the interfacial adhesion between recycled CF and matrix resin is reduced. In the case of commercial virgin CF, the surface of CF is coated with sizing agents for the improvement of interfacial adhesion by a sizing apparatus for continuous fiber. However, it is difficult to apply the manufacturing processes developed for virgin continuous CF, because recycled CF is discontinuous. A complicated process to improve interfacial adhesion is not practical from the viewpoint of manufacturing cost. Therefore, it is critical to develop a simple process to improve interfacial adhesion between various recycled CFs and resin.

Recently, microwave (MW) processes have attracted attention as an innovative curing system for composites composed of CF and thermosetting resins [13,14]. In previous study, the interfacial adhesion between virgin CF and thermosetting epoxy resin was enhanced by MW heating [15]. In this process, CFRP is heated from the inside by the selective heating of CF in the composite. The chemical reaction of epoxy resin and curing agent occurs preferentially on the surface of CF, suggesting that preferential reactions of epoxy resin on the CF interface lead to the suppression of interface delamination, thereby strengthening interfacial adhesion.

The main objective of this study was to clarify the effects of the curing state of epoxy resin and the surface condition of recycle CF on interfacial adhesion between various recycled CFs and epoxy resin as the thermosetting resin matrix heated by MW irradiation. Furthermore, the viscosity and the shrinkage behavior of epoxy resin at the curing temperature were investigated by rheological measurements.

## 2. Materials and Methods

Commercially available CF fabrics (Torayca BT70-20, Toray, Japan) were used in this work. The textile weight of used CF fabrics is 100 g/m^2^. Bisphenol A-type epoxy resin (JER827), bisphenol F-type epoxy resin (JER806), and curing agents (JER cure 3080 or ST11) were purchased from Mitsubishi Chemical Co. Ltd., Tokyo, Japan. These epoxy materials are listed in Table 1. Epoxy resin JER806 was mixed with curing agent ST11 at a mass ratio of 5:3 using a hybrid deformation blender (ARE-310, Thinky, Tokyo, Japan) for 1 min at 2000 rpm. The epoxy resin was penetrated by vacuum-assisted resin transfer molding (VaRTM) to CF fabrics consisting of four plies. The CFRP was pre-cured by maintaining the sample at ambient temperature for 24 h. Post-curing of the composite was performed at 120 °C for 3 h by muffle furnace with an exhaust port (NMF-215B, Masuda Corporation, Chiba, Japan). The volume fiber fraction (V_f_) of the prepared CFRP was measured using JIS K7075 [16]. The V_f_ was 65 vol %. The recycled CF was recovered from prepared CFRP by thermal degradation processing at 500 or 600 °C for 1 h in an air atmosphere. In the recycling process, the heating rate was adjusted to 5 °C/min. Hereafter, virgin CF, recycled CF heated at 500 °C, and recycled CF heated at 600 °C are termed vCF, rCF-500, and rCF-600, respectively.

Thermogravimetric (TG) analyses of CF and CFRP were performed using a thermal analysis instrument (TG-8120, Rigaku, Tokyo, Japan) from 30 °C to 900 °C under natural air of 200 mL/min at a heating rate of 5 °C/min. Furthermore, TG analyses of CFRP were carried out at temperatures of 500 and 600 °C for 3 h at a heating rate of 5 °C/min.

Tensile testing of a single CF was performed using an autograph apparatus (EZ-SX, Shimadzu, Kyoto, Japan) with a load cell of 10 N. Test specimens for tensile testing were prepared by fixing a single CF onto a paper with an adhesive agent. The test specimen was set in the tensile apparatus and the paper was cut into two parts before evaluation. Single-fiber tensile tests were carried out at a gauge length of 25 mm. The cross-head speed was 1.0 mm/min. The tensile strength of the CFs was calculated using the standard Weibull distribution using Equations (1) and (2) [17,18]. The average diameters of the CFs were estimated by field emission scanning electron microscopy (FE-SEM, S-4300, Hitachi, Tokyo, Japan).

(1) $$F\left(\sigma \right)=1-P_{0}=1-exp\left(-{\left(\frac{\sigma }{\gamma }\right)}^{\beta }\right)$$

(2) $$\sigma =\gamma \Gamma \left(1+\frac{1}{\beta }\right)$$

where *β* is the shape parameter, *γ* is the scale parameter, *Γ*(*x*) is the gamma function, and *σ* is the tensile strength.

A single CF was placed in the center of a dog-bone shaped silicon mold. The gauge length of the test specimens for fragmentation testing was fixed at 35 mm. Each end of the CF was fixed with adhesive tape. Epoxy resin JER827 was mixed with curing agent JER cure 3080 at a mass ratio of 2:1 using a hybrid deformation blender for 1 min at 2000 rpm. Epoxy resin was cast into the silicon mold. For this test, the test specimen was irradiated by MW at different times. One was MW-irradiated immediately after resin mixing, and the sample was pre-cured at 23 °C for 24 h. Then, the sample was post-cured at 80 °C for 3 h. The other sample was MW-irradiated after pre-curing at 23 °C for 24 h, and the specimen was post-cured. In this examination, the absorption of MW irradiation of the specimen could not be controlled, as the sample was a single CF. Therefore, the output powers of MW irradiation were controlled at 10, 20, and 30 W. The MW irradiation absorbed by the specimen was calculated by subtracting the reflection power from the output power.

The IFSS between CF and epoxy resin was immediately measured without any conditioning after the preparation of test specimen by a single-fiber fragmentation test. The fragmentation test of the prepared composite was performed using a compact tensile apparatus at a displacement rate of 10 mm/s. The specimen was pulled until the fragments of fiber no more break. During this test, the specimen was observed using a microscope (BX-51, Olympus, Tokyo, Japan) with a digital camera (DP21, Olympus, Tokyo, Japan). The fragment length within the gauge length was measured via image analysis with a calibrated measuring scale. The IFSS between the carbon fiber and epoxy resin was calculated using the following Equation (3) [19,20,21,22]:

(3) $$\tau_{i}=\frac{D\sigma_{f}}{2l_{c}}$$

where *σ_f_* is tensile strength of CF, *D* is diameter of CF, and *l_c_* is the critical fiber length. The critical fiber length (*l_c_*) can be determined using the average fragment length (*l*) by Equation (4):

(4) $$l_{c}=\frac{4}{3}l$$

The average fragment length is the arithmetic mean of the measured fragment lengths at the saturation stage [20]. The IFSS was the average of seven measurements.

**Figure 1:**
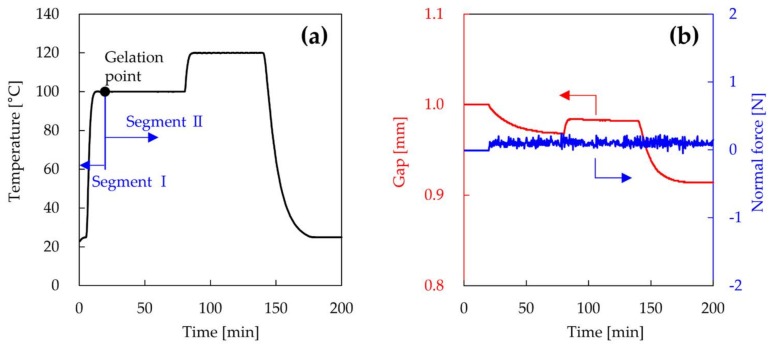
Temperature profile (**a**); gap and normal force (**b**) of epoxy resin at 100 °C setting temperature.

The shrinkage and rheological behavior of the epoxy resin composed of JER827 and JER cure 3080 were measured using a rheometer (MCR102, Anton Paar, Graz, Austria) Disposable aluminum parallel plates with diameters of 25 mm were used in the oscillation mode. The normal force is the amount of force applied by the top plate and is defined as negative when the epoxy resin is in compression. The test was performed in the torque- or strain-controlled mode at a constant angular velocity. Immediately after mixing, the epoxy resin was poured between the plates at room temperature and the gap was set at 1.0 mm. After pouring, the epoxy resin was maintained at 25 °C for 5 min, and heated to 80, 100, and 120 °C until its gelation point was reached. The gelation point of epoxy resin was determined based on the cross-over point of the elastic modulus and loss modulus. After gelation, the sample was heated further at the same temperature for 1 h and post-heated at 120 °C for 1 h. Finally, the sample was cooled to 25 °C. The rheometer was programmed with two segments. In segment I, the gap between the plates was kept constant and the strain was fixed at 15% with an oscillation frequency of 0.2 Hz. When the epoxy resin reached the gelation point, the rheometer was controlled according to segment II. In segment II, the torque was fixed at 0.5 mN with an oscillation frequency of 30 Hz and the normal force was kept constant at 0.1 N. The typical temperature profile, gap, and normal force of epoxy resin at a heating temperature of 100 °C are shown in Figure 1. The rheological behavior of the epoxy resin heated after pre-curing at room temperature was also measured according to the following conditions. The epoxy resin was pre-cured at 23 and 30 °C for 24 h. After pre-curing, the sample was post-heated at 120 °C for 3 h and cooled to 25 °C. The rheometer was also programmed with two segments. In segment a, the gap between the plates was kept constant and the strain was fixed at 1.0% with an oscillation frequency of 1.0 Hz. When the epoxy resin reached the gelation point, the rheometer was controlled by segment b, in which the strain was fixed at 0.02% with an oscillation frequency of 1.0 Hz and a constant normal force of 0.1 N. The linear cure shrinkage *ε_L_* was calculated using the following the Equation (5) [23]:

(5) $$\varepsilon_{L}={\left[\left(1+\nu \right)\left(\frac{\nu }{1-\nu }+1\right)\right]}^{-1}\left(\frac{h_{0}-h}{h_{0}}\right)$$

where *ν* is Poisson’s coefficient of the resin, taken to be 0.35 [24,25], *h* is the gap distance, and *h*_0_ represents the initial gap. Here, this relation assumes that the in-plane strain of the resin is zero.

## 3. Results and Discussion

**Figure 2:**
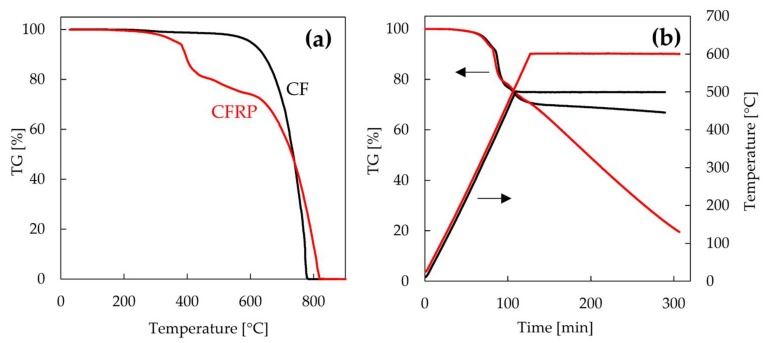
Thermogravimetric (TG) curves of carbon fiber (CF) and carbon fiber-reinforced plastic (CFRP) as a function of temperature (**a**) and CFRP as a function of time under isothermal conditions of 500 and 600 °C (**b**).

Figure 2a shows the TG curves of CF and CFRP as a function of temperature. The weight of CFRP decreased from about 300 °C and decreased greatly at approximately 400 °C. The weight corresponding to the epoxy resin decreased at 300–550 °C. The weight of CFRP decreased further from 600 °C, and the CFRP burned completely at 830 °C. The weight of virgin CF decreased slowly until 600 °C, and virgin CF was destroyed between 600 °C and 800 °C. These results suggest that recycled CF can be recovered from CFRP by thermal degradation at 600 °C or less. Figure 2b shows the TG curves of CFRP as a function of time under isothermal conditions of 500 and 600 °C. The weight losses of CFRP at 500 °C for 0, 1, 2, and 3 h were 26.0, 30.5, 31.6, and 33.1%, respectively. The weight of CFRP decreased slightly by maintaining the temperature at 500 °C. On the other hand, the weight losses of CFRP at 600 °C for 0, 1, 2, and 3 h were 29.4, 46.9, 64.9, and 80.5%, respectively. The weight of CFRP decreased greatly with increasing heating time, indicating that the CF underwent severe decomposition at 600 °C under air. In this study, to investigate the influence of recycled CF with different surface conditions, the temperatures for the thermal degradation recycling process of CFRP were set to 500 and 600 °C for 1 h. After the thermal degradation of bulk CFRP for 1 h in a muffle furnace, the weight losses of CFRP heated at 500 and 600 °C were 32.8 and 45.4%, respectively. These weight losses agreed with the results of TG measurements. The recovery rates of recycled CF heated at 500 and 600 °C estimated from the weight loss were 95.4 and 68.9%, respectively. Therefore, the epoxy resin of CFRP have been completely pyrolyzed in this thermal degradation process.

**Figure 3:**
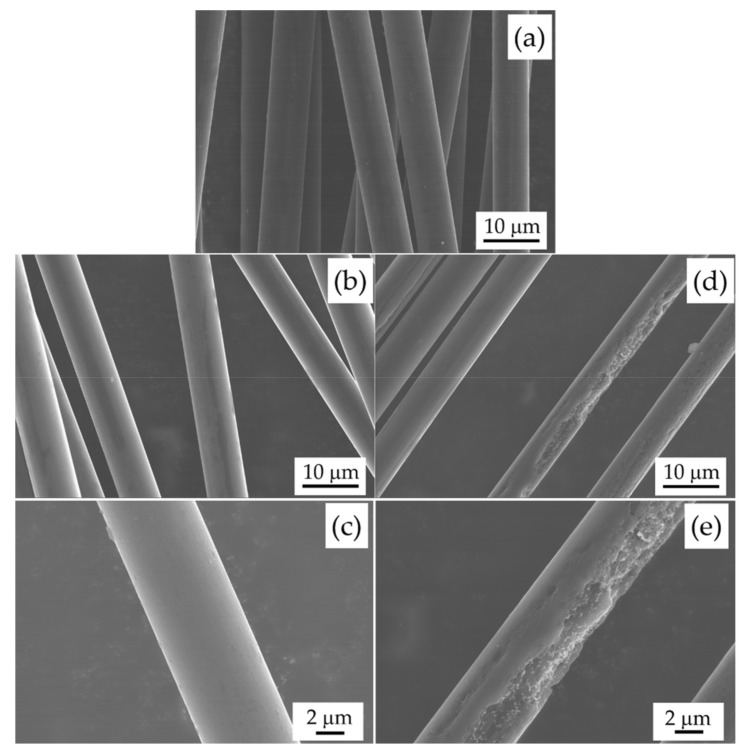
FE-SEM images of vCF (**a**); rCF-500 (**b**,**c**) and rCF-600 (**d**,**e**).

**Figure 4:**
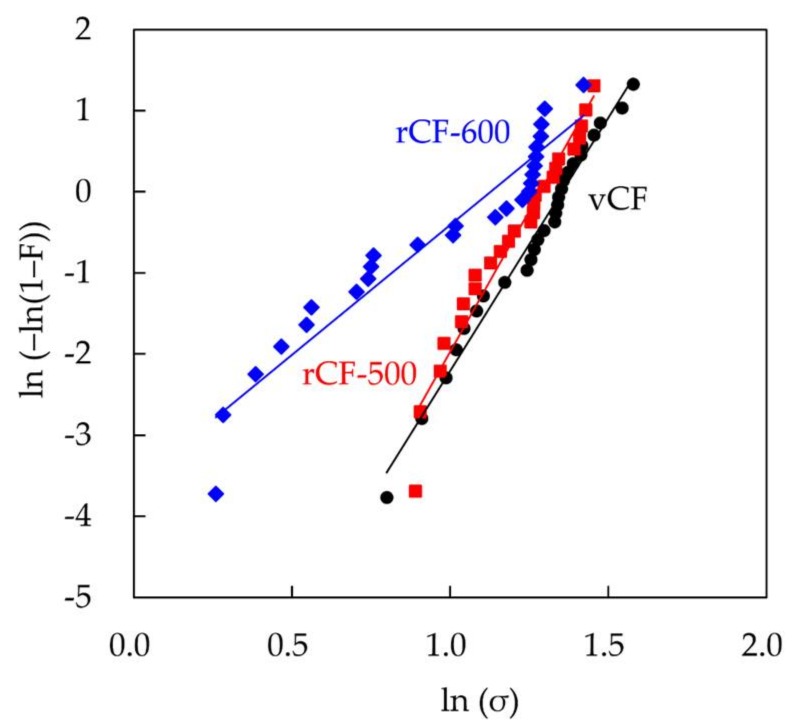
Weibull plots of tensile strengths of vCF (black), rCF-500 (red), and rCF-600 (blue).

Figure 3 shows the FE-SEM images of vCF, rCF-500, and rCF-600. The surface morphology of rCF-500 was almost identical to vCF. On the other hand, the surfaces of several rCF-600 were obviously degraded. The diameters of vCF, rCF-500, and rCF-600 measured from the FE-SEM images were 7.24, 6.87 and 6.21 mm, respectively. The diameter of recycled CF decreased with increasing heating temperature. Figure 4 shows the Weibull plot of the tensile strength of CF. The plot of rCF-600 was not linear and did not conform to the Weibull plot, although the plots of vCF and rCF-500 were almost linear. As shown in Figure 3, rCF-600 contains damaged and undamaged CFs. Because various kinds of defects are introduced into the CFs due to the decomposition of CF, the destruction mode of rCF-600 varies depending on the kinds of the surface defect. Therefore, the rCF-600 did not to conform to the Weibull plot. From these results, the arithmetic mean of the tensile strength of CF with a gauge length of 25 mm was used as the tensile strength of CF. The tensile strengths of vCF, rCF-500, and rCF-600 were 3.60, 3.38, and 2.75 GPa, respectively. The tensile strength of recycled CF decreased with increasing heating temperature.

Table 2 shows the IFSS of the samples cured in a conventional oven. Generally, IFSS is calculated by substituting the fiber ultimate tensile strength at the broken fiber length, which is calculated using a simple weakest-link scaling, into Equation (3) [26,27]. However, rCF-600 did not conform to the Weibull distribution. Therefore, in this study, in order to compare different recycled CFs, the IFSS was calculated by substituting the arithmetic mean of tensile strength of CF with a gauge length of 25 mm into Equation (3). The IFSSs of oven-cured samples composed of vCF, rCF-500, and rCF-600 were 23.8, 18.0, and 14.4 MPa, respectively. The IFSSs of samples prepared with recycled CFs were lower compared with vCF, indicating that the IFSS was decreased by removal of sizing agent in recycling process. Furthermore, the IFSS of the rCF-600 sample was lower compared with rCF-500. This result suggests that the epoxy resin does not impregnate the surface of rCF-600 due to the large defects on the surface.

**Figure 5:**
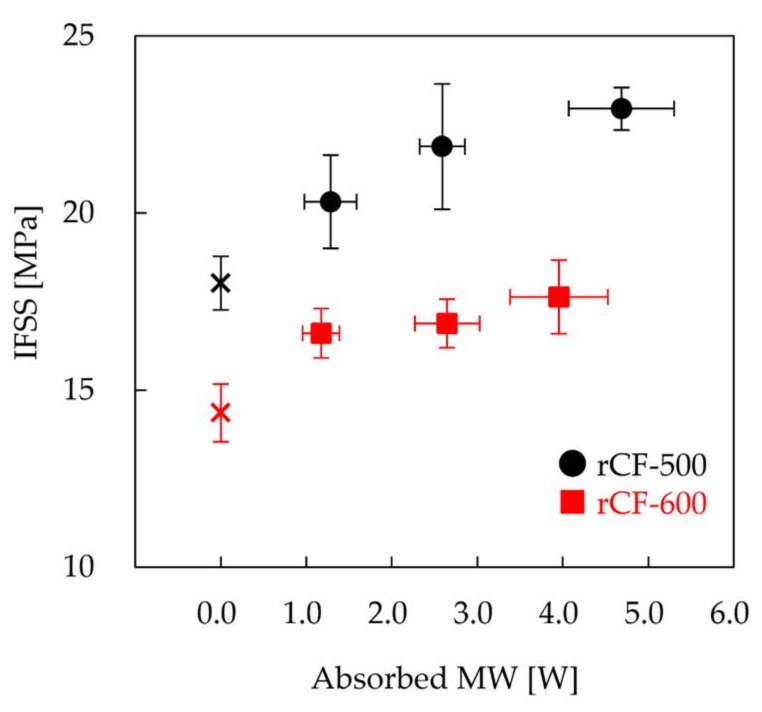
IFSSs of MW-irradiated samples prepared with rCF-500 (black symbol) and rCF-600 (red symbol) as a function of absorbed MW.

**Figure 6:**
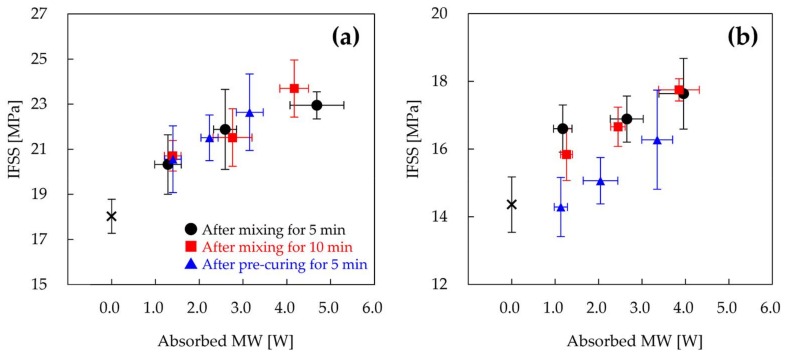
IFSSs of samples prepared with rCF-500 (**a**) and rCF-600 (**b**). The samples were MW-irradiated for 5 min (black circle) or 10 min (red square) after mixing the epoxy resin and curing agent. The sample was MW-irradiated after pre-curing at 23 °C for 24 h (blue triangle).

Figure 5 shows the IFSS of MW-irradiated samples prepared with recycled CF as a function of absorbed MW. Here, the sample was MW-irradiated for 5 min after epoxy resin penetrated the CF and then cured in a conventional oven. In MW irradiation, CF is heated selectively. Because the thermal conductivity of epoxy resin is low, the epoxy resin is cured only at the interface with CF, and the heat of a single CF is not sufficiently transferred to the epoxy resin. Unfortunately, the long duration of MW irradiation results in the thermal degradation of epoxy resin at the interface with CF [15]. Thus, the specimen for the fragmentation test is not cured completely by MW irradiation. In order to evaluate quantitatively the interfacial adhesion between CF and epoxy resin by the fragmentation test, the epoxy resin sample must be cured completely. Therefore, the sample which was cured on the CF interface by MW irradiation was further cured in a conventional oven. The IFSSs of rCF-500 specimens that absorbed MW of 0, 1.3, 2.6, and 4.7 W were 18.0, 20.3, 21.9, and 22.9 MPa, respectively. Additionally, the IFSSs of rCF-600 specimens that absorbed MW of 0, 1.2, 2.6, and 4.0 W were 14.4, 16.6, 16.9, and 17.6 MPa, respectively. The IFSSs of MW-assisted recycled CF samples increased as compared to those without MW irradiation. Furthermore, the IFSSs of samples increased with increasing amounts of absorbed MW. Figure 6 shows the IFSSs of samples prepared by varying the MW irradiation time and the curing state of epoxy resin. The IFSSs of samples MW-irradiated for 10 min were similar to those MW-irradiated for 5 min, indicating that the state of the interface of MW-irradiated samples for 10 min was comparable to those irradiated for 5 min. This result suggests that the epoxy resin on the interface of CF is cured in a short time (within 5 min). The IFSSs of rCF-500 samples MW-irradiated after pre-curing were similar to those MW-irradiated after penetrating. On the other hand, in the case of rCF-600, IFSSs decreased by MW-irradiation after pre-curing. The major difference between rCF-500 and the rCF-600 is the roughness of the surface of recycled CF, suggesting that the roughness of the surface of CF and the curing state of epoxy resin affect interfacial adhesion.

**Figure 7:**
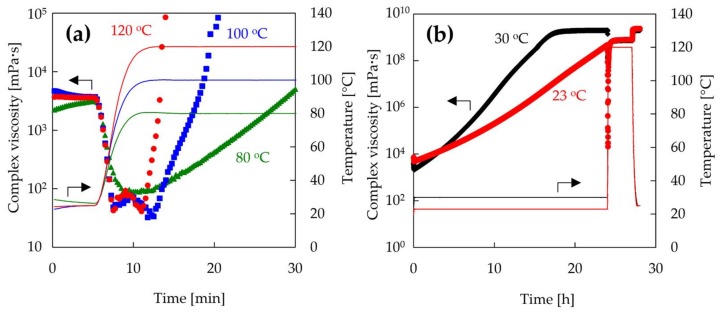
Complex viscosity of epoxy resin at different temperatures as a function of time. The epoxy resin was heated after mixing (**a**) or pre-curing (**b**).

The improvement of the interfacial adhesion between recycled CF and epoxy resin by MW irradiation is now discussed. The improved IFSS is assumed to be mainly derived from the viscosity and shrinkage of the epoxy resin. For the purpose of validating whether the viscosity and the shrinkage of epoxy resin influence the interfacial adhesion, the viscosity variation and the shrinkage behavior of epoxy resin were investigated by rheological measurements. In previous studies, the temperature of CF increased with increasing output power of MW [15]. In fact, the recycled CF was burned off at high output powers of 40 W or more. Because it was very difficult to measure the temperature at the interface during MW irradiation, the viscosity and shrinkage of epoxy resin were measured by changing the heating temperature. Previous work suggests that the resin-fiber interface and the fiber wet-out can be improved by lowering the resin viscosity [14]. Figure 7a shows the complex viscosity of epoxy resin heated at different temperatures after mixing as a function of time. The viscosity of the used epoxy resin decreased considerably by heating at high temperature, following which the epoxy resin became very viscous due to crosslinking. The viscosities of the resins heated at 100 and 120 °C were reduced greatly from 3500 MPa·s to 40 MPa·s, and were lower than that of the resin heated at 80 °C. Furthermore, the viscosity of epoxy resin decreases as the heating rate increases [14]. On the other hand, the viscosities of samples heated after pre-curing varied greatly depending on the temperature of pre-curing, as shown in Figure 7b. The viscosities of the resin heated at 120 °C after pre-curing at 30 and 23 °C were 1.19 × 10^8^ and 2.10 × 10^4^ MPa·s, respectively. The viscosity of the resin pre-heated at 23 °C, which was not gelled for 24 h, decreased greatly. However, the viscosity of the resin pre-heated at 30 °C, which was gelled for 16.5 h, only minimally decreased. Although the viscosity of the resin heated after pre-curing at 23 °C was reduced by heating, the viscosity remained higher than the initial viscosity of the resin. In MW heating, CFs act as a conductor and get abruptly heated due to ionic polarization and dipole orientation, rather than relying on heat transfer through conduction and convection [14,28,29,30]. Therefore, the temperature of the resin at the interface should increase rapidly. The viscosity reduction of the epoxy resin at the interface, based on the high heating rate, would make it easier for the epoxy resin to impregnate the surface of CF in the initial stage of the curing process. Thus, excellent impregnation behavior of epoxy resin by MW irradiation leads to an increase of the reaction efficiency of CF and epoxy resin. In the case of rCF-600, the epoxy resin would be easily impregnated into the rough surface of rCF-600 by irradiating with MW immediately after mixing. On the other hand, MW irradiation after pre-curing might suppress the impregnation of the epoxy resin to the surface of rCF-600. Therefore, the result suggests that the IFSS of rCF-600 samples MW-irradiated after pre-curing would drop due to the lowering of the impregnation based on the high viscosity of the epoxy resin. In the case of the rCF-500, due to the smooth surface of rCF-500, resin impregnation would be unaffected by interfacial adhesion, resulting in IFSSs of MW-irradiated samples after pre-curing to be similar to those irradiated after mixing.

**Figure 8:**
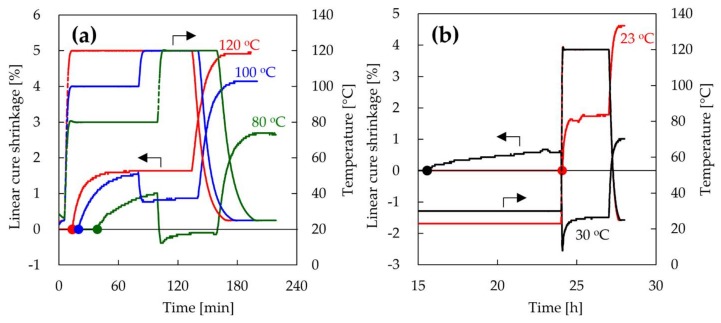
Linear cure shrinkage of epoxy resin as a function of time. Epoxy resin was heated after mixing (**a**) or pre-curing (**b**). The circle symbol is the gelation point of epoxy resin.

**Figure 9:**
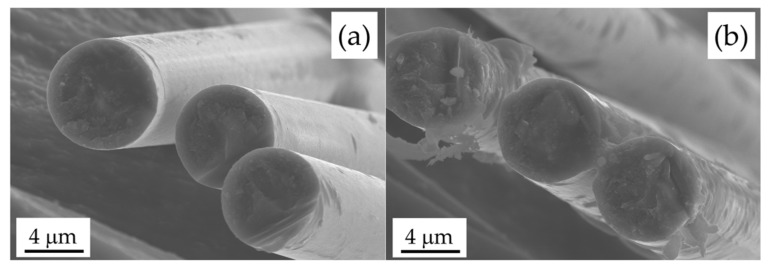
FE-SEM images of rCF-500 (**a**) and rCF-500 (**b**), which were MW-irradiated for 5 min and washed with ethanol to remove unreacted epoxy resin.

The effect of shrinkage of the epoxy resin on interfacial adhesion is also discussed. Figure 8a shows the linear cure shrinkage of epoxy resin after mixing as a function of time. For a curing temperature of 100 °C, the epoxy resin shrunk by curing and then thermally expanded by heating at 120 °C. Finally, the cured epoxy resin thermally shrunk significantly when returning to room temperature. This shrinking behavior of epoxy resin after mixing was observed at all setting temperatures. The linear cure shrinkages of the epoxy resin heated at 80, 100, and 120 °C from the initial stage to when returning to room temperature were 2.65, 4.14, and 4.96%, respectively. The linear cure shrinkage of the epoxy resin increased with increasing initial curing temperature. Figure 8b also shows the linear cure shrinkage of epoxy resin after pre-curing, as a function of time. Furthermore, the linear cure shrinkages of the epoxy resin heated at 120 °C after pre-curing at 23 and 30 °C were 4.62 and 1.01%, respectively. The linear cure shrinkage of the gelled epoxy resin was small even when it was cured at 120 °C. On the other hand, the linear cure shrinkages of the non-gelled samples at 23 °C were almost the same as that of the sample heated at 120 °C immediately after mixing. The pre-curing of epoxy resin before gelation had no effect on the shrinking behavior of the epoxy resin. This result indicates that the temperature during gelation of the epoxy resin is important for the shrinkage of the epoxy resin. These results also showed that the linear cure shrinkage of the sample MW-irradiated after pre-curing were similar to the one MW-irradiated after penetrating. In MW heating, the temperature of the epoxy resin on the CF interface rises rapidly. Furthermore, it is reported that the polarity functional groups with an active oxygen in CF are locally-heated strongly rather than the non-polarity functional groups [31,32]. Therefore, the polarity functional groups can react with the epoxy groups in the epoxy resin and the epoxy resin on the CF interface binds tightly to the CF surface [33,34]. Moreover, in a previous study, the activation energy of the CFRP prepared by MW heating was about one half as compared to the one prepared by conventional heating [35]. Because the reaction rate of epoxy resin is increased by lowering the activation energy, the interfacial adhesion is also improved. Furthermore, the heat generated from CF is diffused outward from the CF, resulting in curing of the epoxy resin which should progress outward from the CF. Figure 9 shows the SEM image of recycled CF, which was MW-irradiated for 5 min and washed with ethanol to remove unreacted epoxy resin. It was confirmed that the epoxy resin was adsorbed on the surface of CF, showing that the epoxy resin was cured from the CF interface. Furthermore, in the MW process, the curing of the epoxy resin outward from the CF interface causes the shrinkage of the epoxy resin along the CF direction. Because the temperature of the epoxy resin near the fiber is high, the shrinkage of the epoxy resin at the interface is significant. As results, the epoxy resin can hug the CF strongly. However, in the case of conventional oven heating, the epoxy resin in the composite is heated from the outside, due to which the chemical reaction of the epoxy resin is initiated from the outside. Because the curing reaction is an exothermic reaction, the reaction of the epoxy resin from the outside is accelerated, and the epoxy resin shrinks from the outside. This shrinking behavior of epoxy resin from the outside causes the delamination of the epoxy resin from CF surface. In fact, the interface of the composite of CF and epoxy resin cured by conventional oven was partially delaminated [15]. Furthermore, the reactivity of the epoxy resin with CF must be lesser than with MW heating, because the temperature of epoxy resin on the CF interface is lower than the setting temperature. Thus, in the conventional oven heating, the interfacial adhesion would decrease. These results suggest that external heating, such as that in an electric oven, requires slow curing at a low temperature for the suppression of shrinkage, whereas MW irradiation can facilitate rapid curing with high-temperature heating. MW irradiation can thus improve the interfacial adhesion between recycled CF and thermosetting resins via mainly shrinkage of epoxy resin along the fiber direction and enhanced impregnation owing to the decrease of viscosity of the resin on the interface.

## 4. Conclusions

To improve the interfacial adhesion between various recycled CFs and epoxy resin by MW irradiation, the IFSSs of composites prepared under different conditions was investigated. In this study, recycled CFs, with or without surface defects, were recovered from CFRP by thermal degradation processes at different temperatures. In the case of the composite MW-irradiated immediately after mixing, the IFSSs of both recycled CFs samples increased with increasing absorbed MW. On the other hand, the IFSS of the composite composed of recycled CF with surface defects did not improve by MW irradiation after pre-curing. We also investigated the curing behavior of epoxy resins by using a rheometer. The viscosity of the epoxy resin decreased greatly by heating immediately after mixing, whereas its reduction was suppressed by heating after pre-curing. The pre-curing of epoxy resin before gelation did not affect the shrinking behavior of epoxy resin. These results suggest that the MW process improved the interfacial adhesion between recycled CF and epoxy resin from two aspects, namely, by enhanced impregnation owing to the decrease of viscosity of the resin on the interface, and the shrinkage of epoxy resin along the fiber direction.

One important consequence of the present work is that the interfacial adhesion between recycled CF and the epoxy resin is significantly affected by the curing state of the epoxy resin before MW irradiation. The interfacial adhesion between various recycled CF and epoxy resin can be improved by MW irradiation immediately after mixing the epoxy resin and curing agent.

## Figures and Tables

**Table 1:** Materials for composites.

Sample	Epoxy		Curing Agent		Mass Ratio of Epoxy to Curing Agent (*w*/*w*)
	Type	EEW ^1^ (g/eq)	Type	AEW ^2^ (KOHmg/g)	
CFRP for recovering recycled CF	JER806 (Bisphenol F)	160–170	ST11 (Amine)	325–360	5/3
Composite for fragmentation test	JER827 (Bisphenol A)	180–190	3080 (Amine)	310–340	2/1

^1^ Epoxy equivalent weight; ^2^ Amine equivalent weight.

**Table 2:** Interfacial shear strength (IFSS) of the samples cured in a conventional oven.

CF	Critical Fiber Length, *l_c_* (mm)	IFSS ^1^ (MPa)	IFSS ^2^ (MPa)
vCF	550 (±34)	46.0 (±3.4)	23.8 (±1.5)
rCF-500	645 (±27)	31.8 (±1.5)	18.0 (±0.8)
rCF-600	597 (±35)	-	14.4 (±0.9)

^1^ IFSS calculated from the fiber ultimate tensile strength at the broken fiber length; ^2^ IFSS calculated from the arithmetic mean of fiber strength of CF with a gauge length of 25 mm.
